# Exploring the Mechanism of *Clostridium autoethanogenum* Protein for Broiler Growth Based on Gut Microbiota and Serum Metabolomics

**DOI:** 10.3390/biology14010029

**Published:** 2025-01-02

**Authors:** Chunqiao Shan, Yan Liu, Sisi Liu, Chuang Li, Chaoxin Ma, Hongmin Yu, Juan Li, Guotuo Jiang, Jing Tian

**Affiliations:** 1 School of Biological Engineering, Dalian Polytechnic University, Dalian 116034, China; shanchunqiao0711@163.com; 2 College of Animal Science and Medicine, Shenyang Agricultural University, Shenyang 110866, China; vicky800206@163.com; 3 Harbin Academy of Agricultural Sciences, Harbin 150028, China; sisi721@163.com; 4 College of Animal Science and Technology, Yangzhou University, Yangzhou 225009, China; 5 Research Quality Control Center, Jiangsu Sanyi Animal Nutrition Technology Co., Ltd., Xuzhou 221300, China; 6 Dalian Sanyi Biotechnology Research Institute, Dalian Sanyi Animal Medicine Co., Ltd., Dalian 116000, China; yuhongmin1214@126.com (H.Y.); 13840943746@163.com (J.L.)

**Keywords:** *Clostridium autoethanogenum* protein, broiler chickens, caecum, microbiome, serum metabolome

## Abstract

Intestinal health is vital for poultry production, and protein plays a key role in intestinal nutrition. The present study used 16S rRNA gene sequencing and serum metabolomics to investigate the effect of CAP on the cecal microflora structure and serum metabolites in 42-day-old broiler chickens. A total of 480 one-day-old Arbor Acres broiler chickens were randomly divided into four treatments with twelve replicates comprising 10 chickens each, evenly divided by sex. The four groups were basal diet group (CAP0), treatment group 1 (CAP2), treatment group 2 (CAP3), and treatment group 3 (CAP4). The broilers in the CAP0 group were fed a basal diet (without CAP), while those in the CAP2, CAP3, and CAP4 groups received diets containing 2%, 3%, and 4% CAP, respectively. Growth performance results showed that dietary CAP supplementation significantly ameliorated the feed conversion rate (FCR) of broilers at 42 days in the CAP3 and CAP4 groups (*p* < 0.05). Microbial results revealed that CAP did not alter the dominant microorganisms in the cecum at the phylum, family, and genus levels. LEfSe analysis showed significantly higher relative abundances of *p_Desulfobacterota, f_Desulfovibrionaceae*, and *g_Ruminococcus* in the CAP3 group compared to the CAP0 and CAP4 groups. Metabolomic analyses indicated that the effect of incorporating CAP into the diet on serum metabolites primarily focused on organic acids and their derivatives, small peptides, amino acid derivatives, and oxidized lipids. The addition of 3% or 4% CAP to the diet can enhance metabolic pathways such as the citrate cycle (TCA cycle) and arginine and proline metabolism. In summary, incorporating CAP into the diet can increase the relative abundance of beneficial bacteria in the cecum and improve the feed conversion efficiency of broilers by enhancing amino acid and energy metabolism.

## Simple Summary

*Clostridium autoethanogenum* protein (CAP), a promising protein feedstock, has garnered mounting attention worldwide, yet the underlying regulatory mechanisms remain to be elucidated. This study aimed to investigate the effects of CAP on the microbial community structure and serum metabolites in the cecum of broiler chickens by 16S rRNA sequencing and metabolomics. The incorporation of CAP into the diet led to an increase in the relative abundance of *p_Bacteriophage*, *f_Desulfovibrioideae*, and *g_Ruminalococcus*, suggesting that CAP does not alter the dominant microorganisms but can increase the relative abundance of beneficial bacteria in the cecum. Furthermore, the dietary incorporation of CAP predominantly influenced serum metabolites, including organic acids, and enhanced metabolic pathways, such as the citrate cycle. These observations suggest that CAP may enhance feed conversion in broilers by promoting amino acid and energy metabolism. A comprehensive understanding of these effects can provide a theoretical foundation for the application of CAP in broiler production, which is imperative for the healthy and sustainable development of the farming industry.

## 1. Introduction

Global demand for food, particularly protein, is expected to increase significantly in the coming decades. This is driven by a rising world population projected to reach approximately 10 billion by 2050, socioeconomic changes such as urbanization in developing countries, and rising income levels [1,2,3,4]. Based on current consumption rates, global demand for animal-derived protein is anticipated to reach 1.25 billion tons per year [5]. The poultry industry has experienced the highest absolute and relative growth rates among the meat production sectors over the past 50 years, attributed to factors such as the lower price of chicken, its convenience, health benefits, and various religious and cultural considerations [6,7]. Consequently, the poultry industry is crucial for fulfilling human demand for animal protein.

The development of the poultry industry is constrained by the high cost of feed resources, which accounts for between 60 and 70 percent of the total cost of production [8]. Protein is the second most crucial nutrient in poultry diets, playing a vital role in the rapid growth of the animals. There are two main types of protein resources used in the poultry industry: plant-based and animal-based feeds. Soybean meal is a particularly favored protein resource in broiler diets due to its high content of crude protein (40% to 48%) and balanced amino acids [9]. However, soybean meal contains anti-nutritional factors, such as phytate and trypsin inhibitors, that negatively impact nutrient utilization [10,11]. Furthermore, the cultivation of soybeans necessitates the availability of arable land and water resources, which are likely to become increasingly scarce in light of the projected growth in the world’s population. The production of single-cell protein (SCP) circumvents the shortcomings associated with low productivity, high water requirements, land use, and environmental impact while containing fewer anti-nutritional factors [12,13]. Existing methods for producing SCP involve culturing microalgae, fungi, and bacteria [14].

Numerous reports currently exist on the use of SCPs in poultry, including *Methylococcus* protein [8], *Chlorella* protein [15], and Methanol-Derived Single-Cell Protein [16]. *Clostridium autoethanogenum* protein (CAP) is a by-product of ethanol production that utilizes CO as the sole carbon source [17]. It contains in excess of 72% crude protein; all essential amino acids similar to fishmeal; and a substantial quantity of carbohydrates, lipids, and vitamins [18]. Recently, the utilization of CAP as a substitute for fishmeal has been extensively employed in aquaculture [19,20,21].

Notably, adding CAP to Cobb broilers’ diets improved growth performance while reducing fat deposition rates [22]. However, no studies have yet explored the application mechanism of CAP in poultry growth promotion using multiple omics approaches. Through the analysis of body metabolites and intestinal microorganisms, the effects of adding CAP to diets on the microscopic level of the body can be profoundly elucidated. Some hallmark metabolites and microorganisms closely related to the body’s growth can be found that can provide an important reference for the healthy and efficient breeding of broiler chickens in the future. Therefore, this paper aims to investigate how CAP enhances broiler growth performance through combined analysis of cecal microbiome and serum metabolomics.

## 2. Materials and Methods

### 2.1. Animals, Diets, and Management

The 480 one-day-old Arbor Acres broilers purchased from Zhenghe Poultry Co., Ltd. (Shangqiu, Henan, China), were randomly divided into four groups: the basal diet group (CAP0), treatment group 1 (CAP2), treatment group 2 (CAP3), and treatment group 3 (CAP4), with each group consisting of 12 replicates, and each replicate containing 10 chickens (equally divided between males and females). The broilers in the CAP0 group were fed a basal diet (devoid of CAP), while the broiler chickens in the CAP2, CAP3, and CAP4 groups were fed diets containing 2%, 3%, and 4% CAP, respectively.

The broiler diets were formulated by the principles of isoenergetic and isonitrogenous nutrition, and the diets for broilers aged between 1 and 3 weeks and between 4 and 6 weeks were found to comply with the technical specifications for low-protein diets for broilers (DB22_T3207-2020). The composition and nutritional levels of the two dietary phases are presented in Table 1 and Table 2, respectively. The temperature and light control were consistent with those described in the previous article [23]. Furthermore, all broilers had free access to food and water.

### 2.2. Growth Performance and Sampling

During the test, the feed intake of broiler chickens was statistically recorded, and the average daily feed intake (ADFI) was calculated. Before the start of the experiment, on days 21 and 42, the animals were fasted for 12 h and weighed, and the average daily gain (ADG) was calculated. The feed conversion ratio (FCR = ADFI/ADG) is calculated based on average daily feed intake and average daily weight gain. On day 42, 6 chickens (half male and half female) from each treatment were randomly selected, and blood was collected from the jugular vein. Subsequently, the broiler chickens were euthanized, and their cervical vertebrae were dislocated and dissected to collect the cecal contents and stored at −80 °C until testing. The blood was centrifuged at 3000 r/min for 10 min at 4 °C to separate the serum and stored at −80 °C.

### 2.3. DNA Extraction and 16S rRNA Sequencing

Genomic DNA was extracted from broiler cecum contents using the Soil FastDNA^®^ Spin Kit (MP Biomedicals, USCAT No. 116,560-200, Omega Bio-tek, Norcross, GA, USA). The V3–V4 region was amplified by PCR, using universal primers [24]. The preparation of sequencing libraries was subsequently performed using the Illumina Library Quantification Kit (Kapa Biosciences, Woburn, MA, USA). The amplicon libraries were then evaluated, and their size distributions and quantities were determined. The libraries were sequenced using the NovaSeq PE250 platform. After the completion of the sequencing process, raw labels were obtained using FLASH software (version 1.2.11) [25]. These labels were then subjected to quality filtering using fastp (version 0.20.0) software [26], and the valid labels were obtained using Vsearch (version 2.15.0). The valid labels were then denoised using QIIME2 (version QIIME2-202006) [27] to obtain ASVs, and these were compared with the database (Silva 138.1).

### 2.4. Bioinformatics Analysis

The richness and diversity of the microflora structure (ACE, Chao1, Shannon, and Simpson) and the dilution curves were performed with the R (V 4.4.0) “vegan” package and visualized with the “ggplot2” package. Beta diversity was assessed using principal coordinate analysis (PCoA), which uses the Bray–Curtis distance algorithm, and visualized using the ggplot2 package in R (V 4.0.2). The microbiota were depicted at the phylum, family, and genus levels using the R package ggplot2. Linear discriminant analysis effect size (LEfSe) analyses were performed by using linear discriminant analysis (LDA) to estimate the effect of the abundance of each component (species) on differential effects [28].

### 2.5. Extraction of Serum Metabolites and Untargeted LC-MS Metabolomics Analysis

The frozen serum samples were removed from the −80 °C refrigerator, thawed, and vortexed for 10 s. Subsequently, 50 μL of the sample was mixed with 300 μL of the 20% acetonitrile/methanol internal standard extract. The mixture was then subjected to thorough vortexing and centrifugated at 8000× *g* for 10 min at 4 °C. Subsequently, 200 μL of the supernatant was placed at −20 °C for 30 min, followed by centrifugation at 8000× *g* for 3 min, at 4 °C. LC-MS metabolomic analyses were conducted at Metware Biotechnology Co. Wuhan, China. The analytical parameters were set as follows: ultra-performance liquid chromatography (UPLC) on a Waters ACQUITY UPLC HSS T3 C18 column (1.8 μm, 2.1 mm × 100 mm) at 40 °C with a flow rate of 0.4 mL/min and an injection volume of 2 μL. The solvent system was water (0.1% formic acid) and acetonitrile (0.1% formic acid), with a gradient program initiated at 95:5 *V*/*V* and concluding at 10:90 *V*/*V*. The gradient program was 95:5 *V*/*V* at 1 min, 10:90 *V*/*V* at 12 min, 95:5 *V*/*V* at 12.1 min, and 95:5 *V*/*V* at 14 min. Quality control (QC) samples were used for each serum sample to assess the reliability and reproducibility of the LC-MS system.

### 2.6. Metabolome Data Processing and Statistical Analysis

The ProteoWizard software (version 3.0.7414) transforms original LC-MS data files into mzML format. The XCMS program is used for peak extraction, alignment, and retention time correction. The “SVR” method is applied to adjust the peak areas. The metabolic features detected in at least 50% of the members in any set of samples are retained. The information for identifying specific metabolites is acquired by searching the self-compiled metabolite database MWDB (Metware Biotechnology Co., Ltd., Wuhan, China) and combining the results with the information obtained from public databases and metDNA.

The variable importance in projection (VIP) values were derived from the orthogonal partial least squares discriminant analysis (OPLS-DA) results, and the *p*-values were obtained from the two-tailed Student’s *t*-test. Before OPLS-DA, the data underwent log2 transformation and were centered on the mean. To prevent overfitting, a permutation test with 200 permutations was conducted. When VIP ≥ 1, FC ≥ 1.5 or ≤0.67, and *p* < 0.05, the metabolite was regarded as having a significant difference between the two groups [29].

The principal component analysis (PCA), partial least squares discriminant analysis (PLS-DA), orthogonal partial least squares discriminant analysis (OPLS-DA), and volcano plots were constructed using SMICA software (V 14.1) [30]. The identified metabolites were annotated with the Compound Human Metabolome Database (HMDB) (https://hmdb.ca/metabolites, accessed on 13 August 2024), and pathway analysis of the detected differentially expressed metabolites was performed using MetaboAnalyst 6.0 (https://www.metaboanalyst.ca, accessed on 13 August 2024).

### 2.7. Growth Performance Data Analysis

The data on the growth performance of the broilers at 42 days of age were collated using Excel 2021. Subsequently, the Shapiro–Wilk test was conducted, followed by one-way ANOVA and Duncan’s method for multiple comparisons using SPSS 26.0. Finally, the data were plotted using GraphPad Prism8.

## 3. Results

### 3.1. Growth Performance

**Figure 1:**
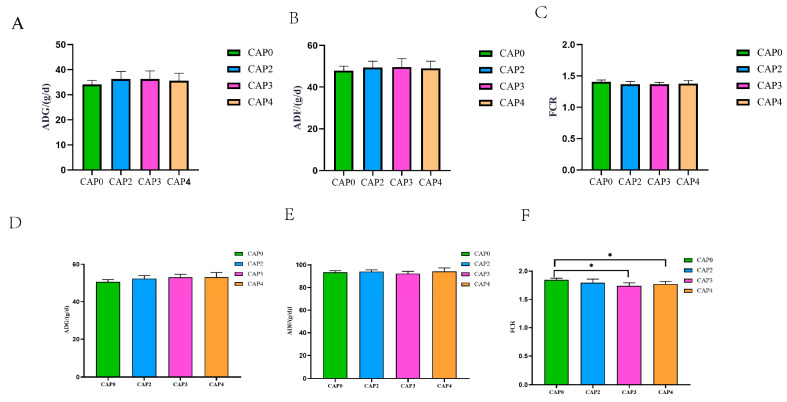
Effect of CAP on the growth performance of broiler chickens at 42 days. (**A**,**D**) ADG. (**B**,**E**) ADF. (**C**,**F**) FCR. (**A**–**C**) Chickens that are 1–21 days old and (**D**–**F**) 1–42 days old. * Indicates a significant difference between the two groups (*p* < 0.05).

The effects of CAP supplementation on the growth performance of broiler chickens at 42 days of age are presented in Figure 1. The results indicated that from 1 to 42 days, the FCR of the CAP3 and CAP4 groups was significantly lower than that of the CAP0 group (*p =* 0.001).

### 3.2. Microbe Composition of Cecum

**Figure 2:**
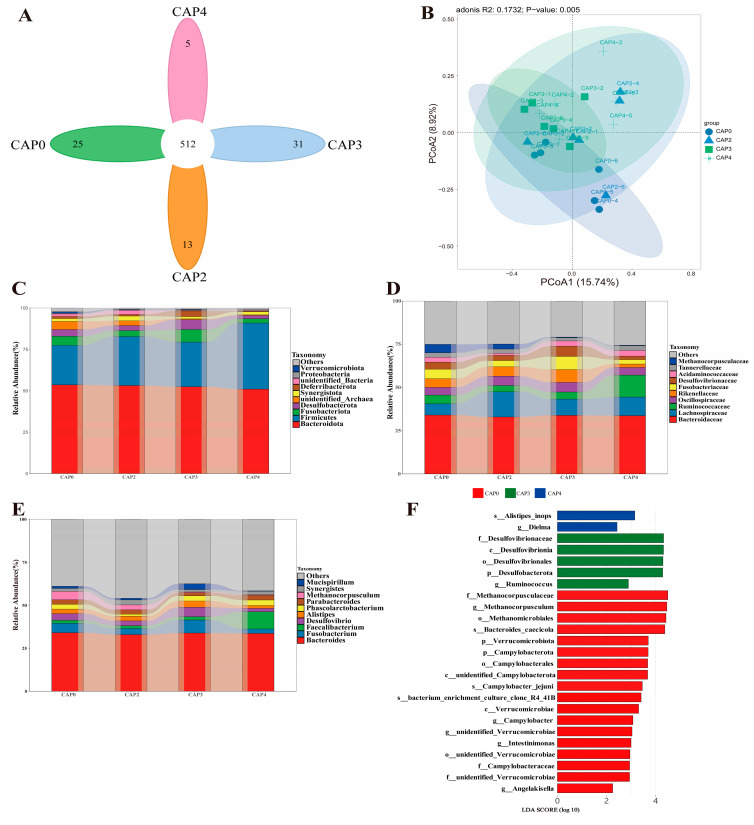
Effect of different CAP intakes on the structure of broiler cecal microbial community. (**A**) Venn diagram. (**B**) Principal coordinate analysis. Top 10 microorganisms at the level of (**C**) phylum, (**D**) family, and (**E**) genus. (**F**) LEfSe analysis.

A total of 2,023,138 sequences were obtained from the cecal microbiome, with 1,379,272 high-quality sequences passing the quality control. Based on a 97% sequence similarity, 878 ASVs were generated, and 586 of them were identified as core ASVs (Figure 2A). The sparse curve showed a flat trend with the increase in the sequence number, suggesting that the sequencing depth was sufficient to cover the majority of bacterial communities in all samples, presented in Supplementary Figure S1. Regarding beta diversity, the Bray–Curtis distance-based PCoA at the ASV level indicated that the separation among groups was not obvious, but the adonis analysis results demonstrated that the addition of CAP significantly affected the microbial community structure in the cecum (*p* = 0.005) (Figure 2B). The addition of CAP did not change the microbial richness (ACE index and Chao index) and diversity (Shannon index and Simpson index) of the broiler cecum (*p* > 0.05), as presented in Supplementary Figure S2. At the phylum level, *Bacteroidota* and *Firmicutes* were the predominant bacterial phyla, accounting for more than 80% of the cecal bacterial community (Figure 2C). At the phylum level, *Bacteroidaceae* emerged as the dominant bacterial family among the four groups (Figure 2D). At the genus level, *Bacteroides* was the dominant bacterial genus within the four groups. The results of the LEfSe analysis (LDA > 2) indicated that *g_Dielma* was significantly enriched in the CAP4 group; *p_Desulfobacterota*, *f_Desulfovibrionaceae*, and *g_Ruminococcus* were markedly enriched in the CAP3 group; and *f_Methanocorpusculaceae*, *g_Methanocorpusculum*, *p_Verrucomicrobiota*, *p_Campylobacterota*, *g_Campylobacter*, *g_unidentified_Verrucomicrobiae*, *g_Intestinimonas*, and *g_Angelakisella* were prominently enriched in the CAP0 group.

### 3.3. Serum Metabolomics Analysis

#### 3.3.1. Sample Quality Control

We further evaluated the stability and reproducibility of the data by using QC samples measured throughout the experimental period. The higher the correlation of QC samples (with R2 approaching 1), the better the data quality. The correlations of QC samples are presented in Supplementary Figure S3. The R2 values were all above 0.99.

#### 3.3.2. Serum Metabolite Analysis

**Figure 3:**
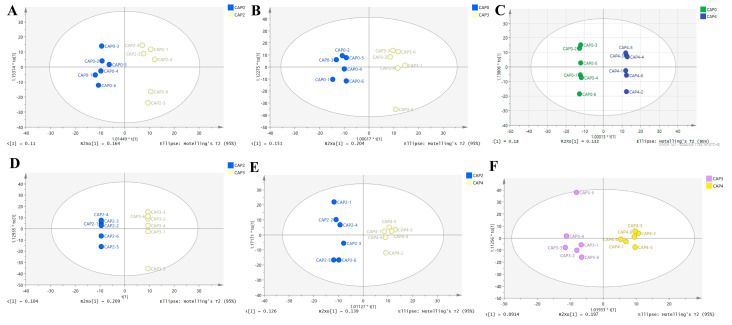
OPLS-DA scores. (**A**) CAP0 and CAP2. (**B**) CAP0 and CAP3. (**C**) CAP0 and CAP4. (**D**) CAP2 and CAP3. (**E**) CAP2 and CAP4. (**F**) CAP3 and CAP4. CAP0: basal diet without CAP. CAP2: diets supplemented with 2% CAP. CAP3: diets supplemented with 3% CAP. CAP4: diets supplemented with 4% CAP.

OPLS-DA (Figure 3A–F) was depicted to distinguish differences between groups, and all samples in the score plots of the six comparisons were within the 95% confidence interval (Hotelling’s T-squared ellipse), with a distinct separation between groups, indicating satisfactory model validity for subsequent difference analysis.

**Figure 4:**
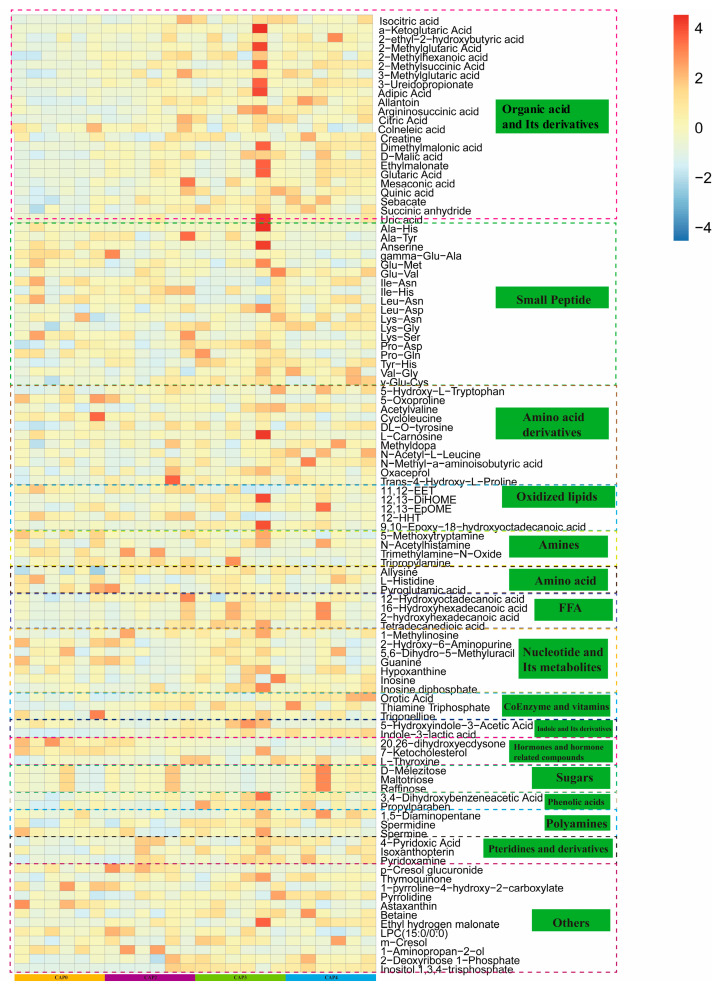
Cluster analysis of differential metabolites. Note: Vertical is the clustering of metabolite components, and horizontal is the grouping, in which yellow represents the CAP0 group, purple represents the CAP2 group, green represents the CAP3 group, and blue represents the CAP4 group. The redder the color of a metabolite, the higher the relative content of this metabolite in the corresponding grouping; the bluer the color of a metabolite, the lower the relative content of this metabolite in the corresponding grouping.

Metabolites with variable importance in projection (VIP) value of ≥1, statistical significance (*p*) ≤ 0.05, and fold change (FC) value of ≥1.5 or ≤0.67 in the orthogonal partial least squares discriminant analysis (OPLS-DA) model were selected as differential metabolites. The largest proportion of metabolites were amino acids and their metabolites, followed by organic acids and their derivatives among all metabolites, as presented in Supplementary Figure S4. The quantities of differential metabolites for the four groups are shown in Supplementary Figure S5. In total, 106 differential metabolites were identified. This analysis particularly highlighted organic acids and their derivatives, small peptides, amino acid derivatives, and oxidized lipids (Figure 4).

A total of 19 core metabolites were altered between the CAP0 and CAP2 groups, with 16 metabolites (Thymoquinone, α-Ketoglutaric Acid, methyl hexanoic acid, etc.) increased significantly, and 3 metabolites (7-Ketocholesterol, Hypoxanthine, and L-Histidine) diminished significantly, as shown in Supplementary Table S1. In total, 39 principal metabolites were altered between the CAP0 and CAP3 groups, with 31 metabolites (Tyr-His, Thiamine Triphosphate, 1-Methylinosine, etc.) increased significantly and 8 metabolites (Ile-Asn, 1-Aminopropan-2-ol, Trimethylamine-N-Oxide, etc.) reduced significantly, as presented in Supplementary Table S2. Fifty-nine principal metabolites exhibited differential expression between the CAP0 and CAP4 groups. Of these, 36 metabolites (inositol 1,3,4-trisphosphate, quinic acid, etc.) demonstrated significant elevation, while 23 metabolites (L-carnosine, 7-ketocholesterol, 20,26-dihydroxyecdysone, etc.) exhibited a significant reduction. These findings are illustrated in Supplementary Table S3.

In the comparison of CAP2 and CAP3 groups, 20 key metabolites changed, with 13 metabolites (including Hypoxanthine, N-Acetylhistamine, and Trigonelline) increased significantly and 7 metabolites (such as 12-HHT, D-Melezitose, and Maltotriose) decreased significantly, as shown in Supplementary Table S4. Between the CAP2 and CAP4 groups, 16 key metabolites were altered, with 8 metabolites (including N-Acetyl-L-Leucine, Val-Gly, and Orotic Acid) increased significantly and 8 metabolites (5-Oxoproline, 7-Ketocholesterol, Colneleic acid, etc.) reduced significantly, referring to Supplementary Table S5. A total of 10 core metabolites exhibited differential expressions between the CAP3 and CAP4 groups. Four metabolites (D-Melezitose and Maltotriose, among others) demonstrated a significant increase, while six metabolites (Spermine and Ala-Tyr, among others) exhibited a significant decrease, as presented in Supplementary Table S6.

**Figure 5:**
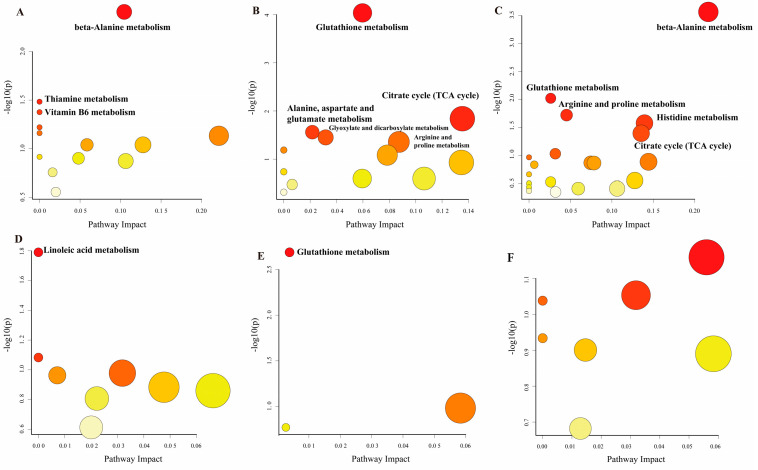
KEGG metabolic pathways. (**A**) CAP0 vs. CAP2. (**B**) CAP0 vs. CAP3. (**C**) CAP0 vs. CAP4. (**D**) CAP2 vs. CAP3. (**E**) CAP2 vs. CAP4. (**F**) CAP3 vs. CAP4. The horizontal coordinate position and size of the bubbles represent the pathway in the topological analysis of the impact factor. The larger the bubble, the greater the impact factor. In the vertical coordinate position and color, the bubbles represent the enrichment analysis of the *p*-value (−log_10_^P^). The smaller the *p*-value, the darker the color, indicating a greater degree of enrichment.

Finally, the KEGG database was utilized for the functional enrichment analysis of the differential metabolites (Figure 5). The beta-Alanine metabolism, Thiamine metabolism, and vitamin B6 metabolism pathways exhibited alterations in the CAP2 group compared to the CAP0 group. In contrast, the CAP3 group demonstrated significant changes in Glutathione metabolism, citrate cycle (TCA cycle), Alanine, aspartate, and glutamate metabolism, as well as glyoxylate and dicarboxylate metabolism, and Arginine and proline metabolism when compared to the CAP0 group. Additionally, the CAP4 group showed significant alterations in beta-Alanine metabolism, Glutathione metabolism, Arginine and proline metabolism, Pyrimidine metabolism, Histidine metabolism, and TCA cycle pathway relative to the CAP0 group. Furthermore, the linoleic acid metabolism pathway was significantly altered in the CAP3 group compared to the CAP2 group. Lastly, the Glutathione metabolism pathway exhibited significant changes in the CAP4 group compared to the CAP2 group.

## 4. Discussion

Broilers are a common type of poultry and serve as a crucial source of protein, providing both meat and eggs for human consumption [31]. However, the development of the broiler industry is significantly constrained by the availability of protein feed resources [32]. In our previous study, we investigated the impact of feed CAP usage on the growth performance of broilers and found that CAP improved broiler growth performance [23]. To the best of our knowledge, the effects of dietary CAP on gut microbiota composition and serum metabolites in broilers remain largely unexplored. Therefore, in this study, we employed 16S high-throughput sequencing technology and metabolomics to analyze the effects of CAP on the gut microbiota structure and serum metabolites of white-feathered broilers.

The gut microbiota plays a pivotal role in nutrient metabolism, host health, and growth performance, particularly within the cecum, a crucial site for intestinal bacterial fermentation [33,34]. Adding CAP to the diet has been proven to enhance the intestinal health of largemouth bass [35]. In this experiment, the predominant phyla among the groups were Firmicutes and Bacteroidetes. Previous studies have demonstrated that members of the Bacteroidetes phylum primarily hydrolyze proteins and degrade carbohydrates, whereas members of the Firmicutes phylum are predominantly involved in energy utilization [36,37]. The application of CAP did not alter the preponderance of the dominant phylum, which aligns with those of previous studies [22]. At the family and genus levels, *Bacteroidaceae* and *Bacteroides*, respectively, were the dominant phyla. Bacteroides are of particular significance in the gut of broilers, playing a pivotal role in the degradation of complex carbohydrates, and they produce hyaluronic acid and acetic acid with plentiful carbon sources, whereas propionic acid is produced when carbon sources are scarce [38,39].

LEfSe analysis revealed that *p_Desulfobacterota*, *f_Desulfovibrionaceae*, and *g_Ruminococcus* were augmented in the CAP3 group. Studies have demonstrated that *g_Ruminococcus* is the predominant bacterial genus in the cecum of broilers and serves as one of the primary producers of acetic acid [40]. Acetic acid plays a crucial role in intestinal energy supply, maintenance of the intestinal mucosal barrier, and regulation of intestinal motility [41]. *g_Ruminococcus* generates short-chain fatty acids (including formic acid, acetic acid, and lactic acid) through glucose metabolism and cellulose degradation. Additionally, it can degrade mucin to provide carbon and energy sources for the host [42]. Consequently, *g_Ruminococcus* plays a vital role in regulating host energy metabolism and enhancing feed conversion efficiency [43,44,45,46]. The escalation in the relative abundance of *g_Ruminococcus* might stem from the employment of 3% CAP in the diet to facilitate digestion and absorption.

Desulfurizing Vibrio species, recognized as sulfate-reducing bacteria, inhibit enzymes implicated in the nicotinamide adenine dinucleotide cycle and ultimately eliminate hydrogen that restricts the production of short-chain fatty acids (SCFAs) [47]. Concurrently, the *Desulfovibrionaceae* family is also a significant source of intestinal pathogens that produce endotoxins. Studies have shown that these bacteria can induce the expression of inflammatory factors in intestinal epithelial cells [48]. Notably, *Desulfovibrio* species exhibit a dual role in host gut health. They not only produce hydrogen sulfide (H_2_S) but also influence the microbial communities within the cecal ecosystem and improve the energy recovery of digestive digesta [44]. Hong Y et al. found that *Desulphurvibrio* bacteria were positively correlated with acetic acid and negatively correlated with metabolic disorders, making them conducive to alleviating non-alcoholic fatty liver disease in mice and, thus, indicating that the bacteria were beneficial to metabolic diseases [49]. In this study, we found that an increase in desulfurizers may contribute to the increased growth performance of CAP-fed broilers. Furthermore, Desulfurization Vibrio is conducive to regulating the microbial community within the cecal ecosystem and enhancing the energy absorption of the digestome in the digestive tract [47]. It can be reasonably deduced that an increase in desulfurizers may contribute to the increased growth performance of CAP-fed broilers.

The metabolome results demonstrated that the impacts of dietary CAP on metabolites in serum were mainly concentrated in organic acid and Its derivatives, small peptides, amino acid derivatives, and oxidized lipids. Additionally, as the amount of CAP increased, more metabolites presented differences, indicating that the quantity of CAP added significantly influenced broiler serum metabolites.

This research has shown that the relative contents of Spermidine and Hydroxyproline were augmented, and flora structure improved when 3% or 4% CAP was incorporated into the diet (as shown in Figure 4). Spermidine can facilitate cell growth and proliferation; enhance immune properties, antioxidant functions, and mitochondrial metabolic function; regulate calcium signaling and respiration; and improve protein homeostasis and chaperone activity [50,51,52,53]. Similar to this study, previous research has shown that supplementation of Spermidine in the diet of Sichuan white goose can enhance the antioxidant capacity of intestinal tissues and regulate the structure of microbial flora [54]. Collagen represents the primary component of connective tissue, and its specific biomarker is hydroxyproline [55]. It can be concluded, therefore, that hydroxyproline affects collagen formation and, as a result, influences skin and bone health [56]. Previous research has found that the quantity of hydroxyproline in the blood of scoliosis chickens is twice that of normal broilers [57]. In the present experiment, with the escalation of CAP content, the blood hydroxyproline content increased, which implies that broilers are at risk of scoliosis. Therefore, the quantity of CAP added in broiler breeding should be further verified to determine whether there is a risk of scoliosis.

Arginine, an essential amino acid for broilers, is a substrate for nitric oxide, proline, and glutamate biosynthesis [58]. The administration of arginine in the diet has been demonstrated to enhance protein synthesis and cellular proliferation while improving feed efficiency [59]. Proline plays a pivotal role in gene transcription and cell differentiation, the scavenging of oxidants, protein synthesis and structure, cell signaling, and bioenergetics [60]. Proline metabolism generates electrons that can enter the mitochondrial electron transport chain, thereby facilitating the production of ATP [61,62]. Furthermore, proline can also be degraded to produce α-ketoglutarate, an intermediate in the citric acid cycle [63]. The metabolic pathways enriched in Spermidine and Hydroxyproline were Arginine and proline metabolism. Spermine is a metabolite of arginine [64]. Therefore, the alterations in spermine levels may be associated with variations in arginine metabolism, specifically with respect to enhanced arginine and proline metabolic pathways. This might be attributed to the higher digestibility of crude protein in CAP, which enhances the utilization of arginine [65]. On the other hand, the quantity of soybean meal employed was reduced, and the content of anti-nutritional factors in the diet formula was decreased.

With the escalation of CAP content, the contents of isocitric acid and citric acid in CAP3 and CAP4 increased significantly, and the corresponding enriched metabolic pathway was the TCA cycle. Isocitric acid and citric acid play pivotal roles in the tricarboxylic acid cycle (TCA) and are vital intermediates in the metabolism of sugar, fat, and amino acids. These compounds have significant functions in the growth and development of animals. Citric acid, a prevalent additive in human food and animal feed, is extensively utilized. Citric acid, the initial intermediate metabolite of the TCA cycle, has been demonstrated to enhance the balance of intestinal flora, fortify immunity, and improve nutrient digestibility, thereby promoting the growth of broilers [66,67]. The TCA cycle is accountable for the complete oxidation of acetyl-CoA from pyruvate under aerobic conditions. Secondly, TCA cycle intermediates are requisite for the biosynthesis and catabolism of several amino acids [68]. The intensified TCA cycle implies that more energy can be provided. The intensification of the TCA cycle might be attributed to the substantial amount of α-ketoglutarate produced by arginine and proline during metabolism entering the TCA cycle. Therefore, adding CAP to the diet might have enhanced the energy digestibility. In the future, the major metabolites enriched in the metabolic pathway and the signature microorganisms in the gut can be further validated by cellular and feeding experiments for the healthy and efficient breeding of broilers.

## 5. Conclusions

In conclusion, incorporating 3% CAP into broiler diets improved the feed conversion efficiency by 42 days of age. This enhancement may be linked to a higher relative abundance of *p_Desulfobacterota*, *f_Desulfovibrionaceae*, and *g_Ruminococcus* in the cecum. Additionally, there was an up-regulation of arginine and proline metabolism pathways and the tricarboxylic acid cycle that increased energy utilization efficiency. However, an increased risk of scoliosis in broilers was observed with higher levels of CAP. Therefore, it is recommended that the maximum addition of CAP to broiler diets should not exceed 3%.

## Figures and Tables

**Table 1:** Diet composition and nutritional level of broiler chickens for 1–3 weeks (air-dried basis) %.

Items	Groups ^①^			
	CAP0	CAP2	CAP3	CAP4
Raw material				
Corn	62.12	62.45	61.95	62.28
Wheat bran	0	2.9	5	6.2
Soybean meal	28	24.8	22.73	21.2
Puffed soybean powder	5	3	2.5	1.5
CAP	0	2	3	4
Soybean oil	1	1	1	1
CaHPO_4_·2H_2_O	1.5	1.55	1.52	1.53
Stone powder	1.38	1.37	1.41	1.43
NaCl	0.3	0.3	0.3	0.3
Mineral premix ^②^	0.1	0.1	0.1	0.1
Vitamin premix ^③^	0.03	0.03	0.03	0.03
Phytase (powder)	0.02	0.02	0.02	0.02
Choline chloride	0.1	0.1	0.1	0.1
Lysine	0.18	0.14	0.12	0.1
Methionine	0.2	0.17	0.16	0.15
Threonine	0.07	0.07	0.06	0.06
Total	100	100	100	100
Nutrient content				
CP, %	19.56	19.55	19.56	19.57
CF, %	2.94	2.89	2.90	2.87
Ash, %	4.45	5.48	4.15	4.01
ME, Kcal/kg ^④^	2973	2973	2972	2974
Ca, %	0.92	0.91	0.91	0.92
P-total, %	0.62	0.62	0.62	0.62
P-available, %	0.36	0.37	0.36	0.36
Lys, %	1.15	1.15	1.15	1.15
Met, %	0.50	0.50	0.50	0.50
Thr, %	0.80	0.80	0.80	0.80
Trp, %	0.22	0.21	0.21	0.20
Val, %	0.90	0.93	0.94	0.96

Note: ① The usage of CAP in CAP0, CAP2, CAP3, and CAP4 is 0%, 2%, 3%, and 4%, respectively. ② Mineral premix provides Cu 8 mg, Fe 80 mg, Mn 80 mg, Zn 80 mg, I 0.5 mg, and Se 0.3 mg per kilogram of basal feed. ③ Vitamin premix provides per kilogram of basic feed: VA 8000 IU, VD3 1000 IU, VE 20 IU, VK3 0.5 mg, VB1 2 mg, VB2 8 mg, VB6 3 mg, VB12 0.01 mg, niacin 35 mg, folic acid 0.55 mg, pantothenic acid 10 mg, and biotin 0.15 mg. ④ ME: metabolizable energy. The nutrient level is the calculated value.

**Table 2:** Diet composition and nutritional level of broiler chickens for 4–6 weeks (air-dried basis).

Items	Groups ^①^			
	CAP0	CAP2	CAP3	CAP4
Raw material				
Corn	67.6	68.2	67.19	66.13
Wheat bran	0.00	2.65	5.30	8.07
Soybean meal	23.43	20.53	17.90	15.20
Puffed soybean powder	3.3.	1.00	1.00	1.00
CAP	0.00	2.00	3.00	4.00
Soybean oil	2.00	2.00	2.00	2.00
CaHPO_4_·2H_2_O	1.20	1.20	1.20	1.20
Stone powder	1.43	1.45	1.47	1.47
NaCl	0.30	0.30	0.30	0.30
Mineral premix ^②^	0.10	0.10	0.10	0.10
Vitamin premix ^③^	0.03	0.03	0.03	0.03
Phytase (powder)	0.02	0.02	0.02	0.02
Choline chloride	0.10	0.10	0.10	0.10
Lysine	0.18	0.13	0.11	0.10
Methionine	0.18	0.16	0.15	0.14
Threonine	0.12	0.11	0.11	0.11
Tryptophan	0.01	0.02	0.02	0.03
Total	100	100	100	100
Nutrient content				
CP, %	17.50	17.50	17.50	17.50
CF, %	2.67	2.61	2.65	2.69
Ash, %	4.97	5.02	5.06	5.10
ME, Kcal/kg ^④^	3062	3062	3062	3062
Ca, %	0.85	0.85	0.85	0.85
P-total, %	0.55	0.54	0.54	0.55
P-available, %	0.30	0.30	0.30	0.30
Lys, %	1.00	1.00	1.00	1.00
Met, %	0.46	0.46	0.46	0.46
Thr, %	0.76	0.76	0.76	0.76
Trp, %	0.20	0.20	0.20	0.20
Val, %	0.80	0.82	0.84	0.85

Note: ① The usage of CAP in CAP0, CAP2, CAP3, and CAP4 is 0%, 2%, 3%, and 4%, respectively. ② Mineral premix provides Cu 8 mg, Fe 80 mg, Mn 80 mg, Zn 80 mg, I 0.5 mg, and Se 0.3 mg per kilogram of basal feed. ③ Vitamin premix provides per kilogram of basic feed: VA 8000 IU, VD3 1000 IU, VE 20 IU, VK3 0.5 mg, VB1 2 mg, VB2 8 mg, VB6 3 mg, VB12 0.01 mg, niacin 35 mg, folic acid 0.55 mg, pantothenic acid 10 mg, and biotin 0.15 mg. ④ ME: metabolizable energy. The nutrient level is the calculated value.

## Data Availability

The data presented in this study are available in the article.

## Supplementary Materials

The following supporting information can be downloaded at https://www.mdpi.com/article/10.3390/biology14010029/s1, Supplementary Figure S1: Sample dilution curve; Supplementary Figure S2: Alpha diversity; Supplementary Figure S3: Metabolome quality control; Supplementary Figure S4: The proportion of different categories of metabolites; Supplementary Figure S5: The number of different metabolites between groups; Table S1: Metabolites significantly different between CAP0 and CAP2; Table S2: Metabolites significantly different between CAP0 and CAP3; Table S3: Metabolites significantly different between CAP0 and CAP4; Table S4: Metabolites significantly different between CAP2 and CAP3; Table S5: Metabolites significantly different between CAP2 and CAP4; Table S6: Metabolites significantly different between CAP3 and CAP4.
