# Supplementary material for: Exploring the Mechanism of Clostridium autoethanogenum Protein for Broiler Growth Based on Gut Microbiota and Serum Metabolomics
Source: Biology (Basel). 2025 Jan 2;14(1):29. doi: 10.3390/biology14010029 (PMC11762677; doi:10.3390/biology14010029)
Supplement: Supplementary file 1 [file biology-14-00029-s001.zip › biology-3378707-supplementary.pdf]

# Exploring the mechanism of *Clostridium autoethanogenum* protein for broiler growth based on gut microbiota and serum metabolomics

Chunqiao Shan[1†], Yan Liu[2†], Sisi Liu[3] Chuang Li[4], Chaoxin Ma[5], Hongmin Yu[6], Juan Li[6], Guotuo Jiang[2*]and Jing Tian[1*]

[1]School of Biological Engineering, Dalian Polytechnic University, Dalian, 116034, China

[2]College of Animal Science and Medicine, Shenyang Agricultural University, Shenyang, 110866, China

[3]Harbin Academy of Agricultural Sciences, Harbin, 150028, China

[4]College of Animal Science and Technology, Yangzhou University, Yangzhou, 225009, China

[5]Research Quality Control Center, Jiangsu Sanyi Animal Nutrition Technology Co., Ltd., Xuzhou, 221300, China

[6]Dalian Sanyi Biotechnology Research Institute, Dalian Sanyi Animal Medicine Co., Ltd., Dalian 116000, China

[†] These authors contributed equally to this work and share first authorship

[*]Correspondence: jgt600@126.com; tianjing@dlpu.edu.cn

# Supplemental materials

## summary

The supporting information includes·.5 supplementary figures and 6 supplementary table.

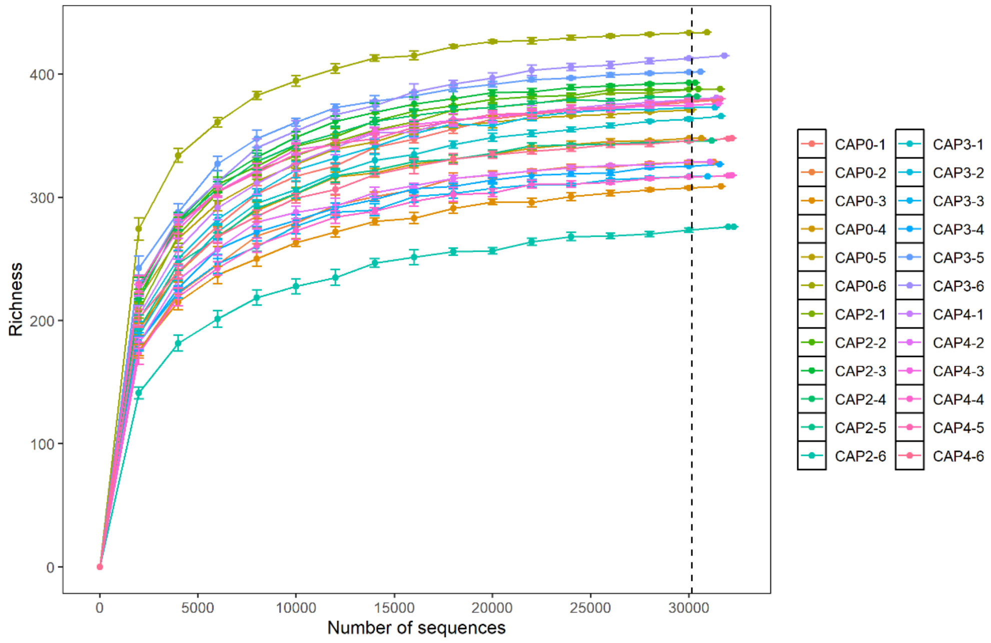


Supplementary figure S1

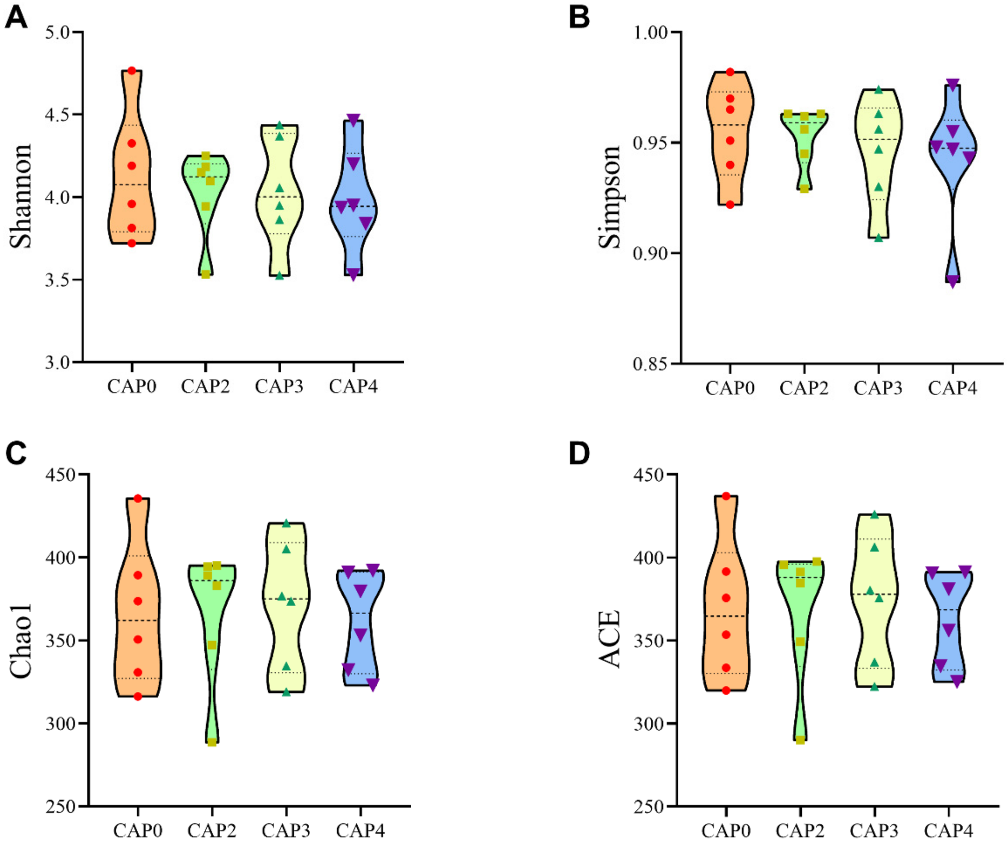


Supplementary figure S2. Alpha diversity

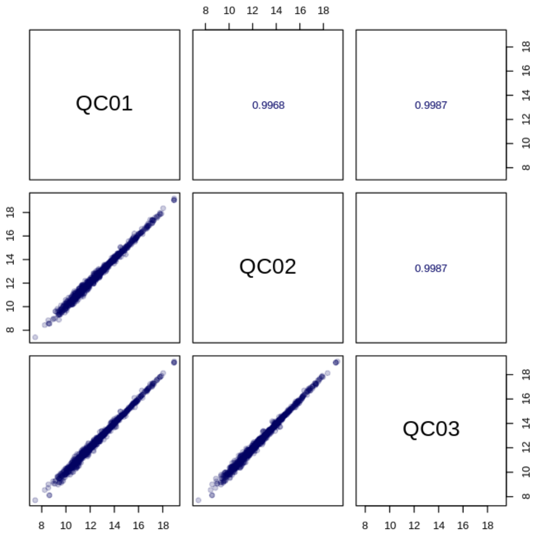


Supplementary figure S3

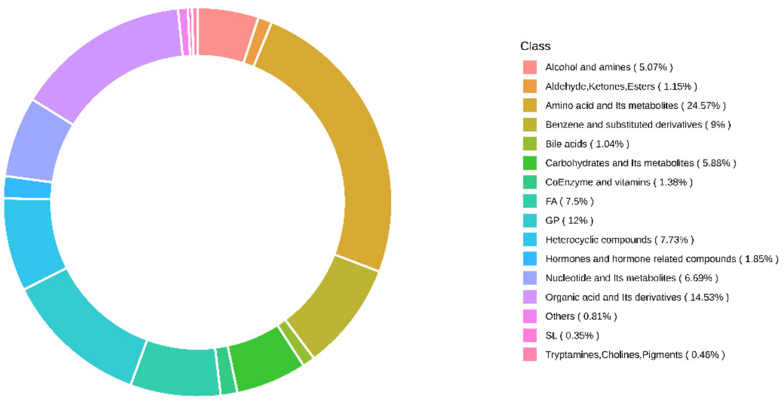


Supplementary figure S4. The proportion of different categories of metabolites

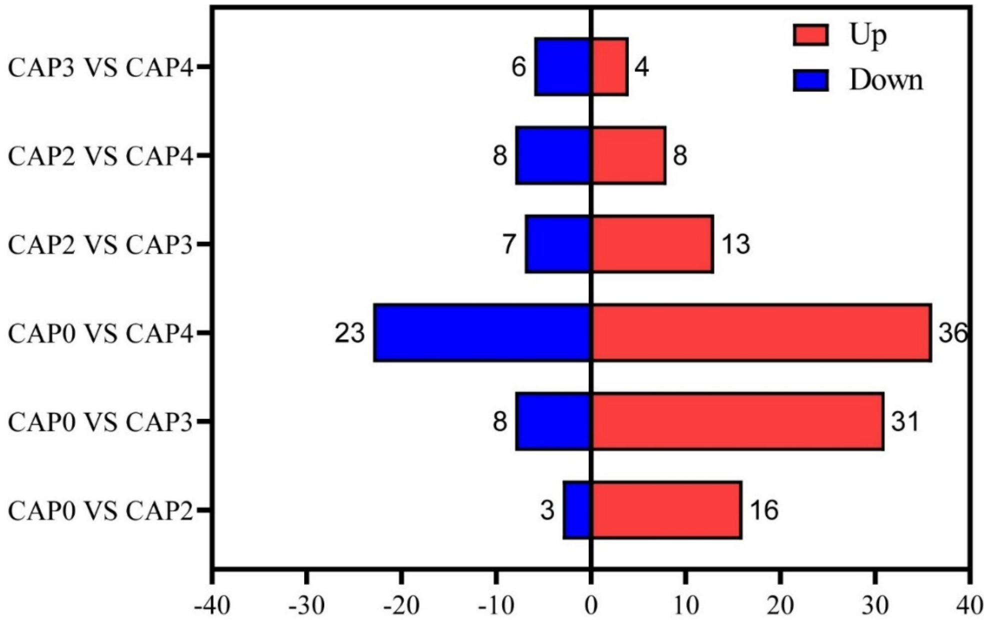


Supplementary figure S5. The number of different metabolites between groups

Supplementary table S1. CAP0 vs CAP2

| Table S1: Metabolites significantly different between CAP0 and CAP2 | | | | |
|---|---|---|---|---|
| CAP0 VS CAP2 | P value | Fold change | VIP | Trend |
| 4-Pyridoxic Acid | 0.0428005 | 2.14243 | 1.60191 | up |
| D-Malic acid | 0.0125049 | 2.0949 | 1.77544 | up |
| α-Ketoglutaric Acid | 0.00225919 | 2.02673 | 2.03385 | up |
| Sebacate | 0.00642277 | 2.02512 | 1.80326 | up |
| Thiamine Triphosphate | 0.000627232 | 2.02478 | 2.25177 | up |
| Allysine | 0.0150695 | 1.905 | 1.78627 | up |
| Indole-3-lactic acid | 0.00217704 | 1.81316 | 2.13299 | up |
| Thymoquinone | 0.0302512 | 1.74026 | 1.50176 | up |
| 2-Methylsuccinic Acid | 0.00786274 | 1.67068 | 2.00892 | up |
| 3-Ureidopropionate | 0.00786274 | 1.67068 | 2.00892 | up |
| Dimethylmalonic acid | 0.00786274 | 1.67068 | 2.00892 | up |
| Ethyl hydrogen malonate | 0.00786274 | 1.67068 | 2.00892 | up |
| Ethylmalonate | 0.00786274 | 1.67068 | 2.00892 | up |
| Glutaric Acid | 0.00786274 | 1.67068 | 2.00892 | up |
| 2-Methylhexanoic acid | 0.0133951 | 1.59212 | 1.82152 | up |
| 12-Hydroxyoctadecanoic acid | 0.0478249 | 1.5599 | 1.51936 | up |
| 7-Ketocholesterol | 0.00477562 | 0.663669 | 2.01053 | down |
| L-Histidine | 0.0187896 | 0.601604 | 1.74473 | down |
| Hypoxanthine | 0.0469381 | 0.407952 | 1.62206 | down |

Supplementary table S2. CAP0 vs CAP3

| Table S2: Metabolites significantly different between CAP0 and CAP3 | | | | |
|---|---|---|---|---|
| CAP0 VS CAP3 | Fold change | P value | VIP | Trend |
| Inositol 1,3,4-trisphosphate | 5.87861 | 0.000518615 | 1.88881 | up |
| Tetradecanedioic acid | 3.33871 | 0.0115959 | 1.50892 | up |
| Inosine diphosphate | 2.96002 | 0.0371112 | 1.42285 | up |
| D-Malic acid | 2.7342 | 0.0117124 | 1.64095 | up |
| Tyr-His | 2.59149 | 0.000147595 | 1.95304 | up |
| Quinic acid | 2.55894 | 0.0345169 | 1.43312 | up |
| Pyridoxamine | 2.35105 | 0.00628496 | 1.64368 | up |
| Citric Acid | 2.31316 | 0.012447 | 1.63682 | up |
| Isocitric acid | 2.31316 | 0.012447 | 1.63682 | up |
| 1-Methylinosine | 2.1197 | 0.0173362 | 1.70772 | up |
| Sebacate | 2.09575 | 0.0171141 | 1.44053 | up |
| Leu-Asp | 2.09297 | 0.0247616 | 1.49107 | up |
| Indole-3-lactic acid | 2.07649 | 0.000179188 | 1.96488 | up |
| 3,4-Dihydroxybenzeneacetic Acid | 2.0616 | 0.0383342 | 1.51547 | up |
| Allysine | 1.9693 | 0.00698082 | 1.67808 | up |
| Mesaconic acid | 1.95412 | 0.048428 | 1.27206 | up |
| Thiamine Triphosphate | 1.9278 | 0.0105502 | 1.46802 | up |
| Isoxanthopterin | 1.91173 | 0.0387192 | 1.50645 | up |
| 16-Hydroxyhexadecanoic acid | 1.89224 | 0.0103901 | 1.40321 | up |
| 2-hydroxyhexadecanoic acid | 1.89224 | 0.0103901 | 1.40321 | up |
| 3-Methylglutaric acid | 1.87067 | 0.00158416 | 1.876 | up |
| Trans-4-Hydroxy-L-Proline | 1.82605 | 0.00611326 | 1.68504 | up |
| Oxaceprol | 1.81528 | 0.0422617 | 1.47357 | up |
| Argininosuccinic acid | 1.73502 | 0.024625 | 1.51717 | up |
| Pro-Asp | 1.6999 | 0.0148859 | 1.56675 | up |
| Spermidine | 1.69201 | 0.0208804 | 1.477 | up |
| γ-Glu-Cys | 1.61615 | 0.00367788 | 1.75051 | up |
| Succinic anhydride | 1.56585 | 0.0179269 | 1.5785 | up |
| m-Cresol | 1.55857 | 0.0444806 | 1.35279 | up |
| Propylparaben | 1.52305 | 0.0171305 | 1.62991 | up |
| Acetylvaline | 1.51037 | 0.0257528 | 1.46207 | up |
| gamma-Glu-Ala | 0.607549 | 0.0102669 | 1.61038 | down |
| 5-Oxoproline | 0.602108 | 0.0173263 | 1.4549 | down |
| Ile-Asn | 0.577737 | 0.0283415 | 1.36433 | down |
| Leu-Asn | 0.577737 | 0.0283415 | 1.36433 | down |
| 7-Ketocholesterol | 0.565604 | 0.0465448 | 1.54452 | down |
| 20,26-dihydroxyecdysone | 0.394351 | 0.00145685 | 1.86934 | down |
| 1-Aminopropan-2-ol | 0.324955 | 0.0263191 | 1.53681 | down |
| Trimethylamine-N-Oxide | 0.324955 | 0.0263191 | 1.53681 | down |

Supplementary table S3. CAP0 vs CAP4

| Table S3: Metabolites significantly different between CAP0 and CAP4 | | | | |
|---|---|---|---|---|
| CAP0 VS CAP4 | P value | Fold change | VIP | Trend |
| Inositol 1,3,4-trisphosphate | 4.33E-07 | 6.62996 | 1.93176 | up |
| N-Methyl-α-aminoisobutyric acid | 0.0487319 | 2.59494 | 1.31988 | up |
| Indole-3-lactic acid | 2.62E-07 | 2.54369 | 1.9303 | up |
| Quinic acid | 0.00504539 | 2.54092 | 1.50819 | up |
| Sebacate | 0.0172911 | 2.36916 | 1.37208 | up |
| Adipic Acid | 0.00116555 | 2.3587 | 1.67541 | up |
| L-Thyroxine | 0.0158601 | 2.21869 | 1.41028 | up |
| Thymoquinone | 0.00178214 | 2.21417 | 1.63021 | up |
| Pyridoxamine | 0.0235368 | 2.1695 | 1.39446 | up |
| Thiamine Triphosphate | 0.024139 | 2.11836 | 1.27452 | up |
| Uric acid | 0.0351974 | 2.09604 | 1.23237 | up |
| Tyr-His | 0.0043905 | 2.09314 | 1.55935 | up |
| 2-ethyl-2-hydroxybutyric acid | 0.0442528 | 2.08505 | 1.26155 | up |
| 2-Methylsuccinic Acid | 0.00215997 | 2.06534 | 1.63282 | up |
| 3-Ureidopropionate | 0.00215997 | 2.06534 | 1.63282 | up |
| Dimethylmalonic acid | 0.00215997 | 2.06534 | 1.63282 | up |
| Ethyl hydrogen malonate | 0.00215997 | 2.06534 | 1.63282 | up |
| Ethylmalonate | 0.00215997 | 2.06534 | 1.63282 | up |
| Glutaric Acid | 0.00215997 | 2.06534 | 1.63282 | up |
| Orotic Acid | 0.000799172 | 2.04361 | 1.69583 | up |
| Citric Acid | 0.0215452 | 2.01152 | 1.29834 | up |
| Isocitric acid | 0.0215452 | 2.01152 | 1.29834 | up |
| 2-Methylglutaric Acid | 0.00360148 | 1.99201 | 1.58287 | up |
| Mesaconic acid | 0.0219404 | 1.9602 | 1.29282 | up |
| Trans-4-Hydroxy-L-Proline | 0.0113489 | 1.91736 | 1.39516 | up |
| Allysine | 0.0182488 | 1.81536 | 1.47557 | up |
| Allantoin | 0.00523972 | 1.66337 | 1.5503 | up |
| Lys-Gly | 0.00661663 | 1.63452 | 1.51676 | up |
| Pyrrolidine | 0.00142427 | 1.58626 | 1.6901 | up |
| Glu-Val | 0.00958981 | 1.57784 | 1.42402 | up |
| 3,4-Dihydroxybenzeneacetic Acid | 0.0389435 | 1.56696 | 1.29423 | up |
| 5-Hydroxy-L-Tryptophan | 0.0330689 | 1.56395 | 1.26628 | up |
| Creatine | 0.0272698 | 1.52707 | 1.46834 | up |
| Methyldopa | 0.0441861 | 1.51369 | 1.32765 | up |
| 2-Deoxyribose 1-Phosphate | 0.00561155 | 1.50831 | 1.61263 | up |
| DL-O-tyrosine | 0.0259123 | 1.50123 | 1.37091 | up |
| Lys-Ser | 0.0169454 | 0.65869 | 1.39091 | down |
| LPC(15:0/0:0) | 0.028714 | 0.635594 | 1.29844 | down |
| Spermine | 0.0137321 | 0.629974 | 1.48227 | down |
| Glu-Met | 0.0194179 | 0.61698 | 1.36151 | down |
| Pyroglutamic acid | 0.0437683 | 0.610906 | 1.32051 | down |

| | | | | |
|---|---|---|---|---|
| Astaxanthin | 0.00852676 | 0.555889 | 1.4906 | down |
| 2-Hydroxy-6-Aminopurine | 0.0229777 | 0.543653 | 1.41869 | down |
| Guanine | 0.0229777 | 0.543653 | 1.41869 | down |
| 5-Hydroxyindole-3-Acetic Acid | 0.0212555 | 0.538226 | 1.34923 | down |
| 5-Oxoproline | 0.00545501 | 0.516367 | 1.57662 | down |
| gamma-Glu-Ala | 0.00256772 | 0.515767 | 1.63895 | down |
| Cycloleucine | 0.00682486 | 0.474283 | 1.55229 | down |
| 1-pyrroline-4-hydroxy-2-carboxylate | 0.0338948 | 0.474024 | 1.29956 | down |
| 5-Methoxytryptamine | 0.0206372 | 0.472243 | 1.46944 | down |
| Pro-Gln | 0.0109835 | 0.439888 | 1.43947 | down |
| L-Carnosine | 0.049208 | 0.398072 | 1.28143 | down |
| 7-Ketocholesterol | 1.17E-05 | 0.387764 | 1.88314 | down |
| 20,26-dihydroxyecdysone | 0.00113278 | 0.37632 | 1.66732 | down |
| Ala-His | 0.031816 | 0.356786 | 1.34777 | down |
| 1-Aminopropan-2-ol | 0.0137465 | 0.333807 | 1.45861 | down |
| Trimethylamine-N-Oxide | 0.0137465 | 0.333807 | 1.45861 | down |
| Anserine | 0.0147601 | 0.330733 | 1.48375 | down |
| p-Cresol glucuronide | 0.0291203 | 0.31031 | 1.37704 | down |

Supplementary table S4. CAP2 vs CAP3

| Table S4: Metabolites significantly different between CAP2 and CAP3 | | | | |
|---|---|---|---|---|
| CAP2 VS CAP3 | P value | Fold change | VIP | Trend |
| 9,10-Epoxy-18-hydroxyoctadecanoic acid | 0.0276936 | 3.93639 | 1.58689 | up |
| 12,13-DiHOME | 0.0373273 | 3.87723 | 1.53304 | up |
| Hypoxanthine | 0.0164723 | 3.35771 | 1.67441 | up |
| N-Acetylhistamine | 0.0347772 | 2.14303 | 1.55611 | up |
| Trigonelline | 0.0214007 | 2.03075 | 1.63147 | up |
| Tyr-His | 0.00658276 | 1.88104 | 1.75302 | up |
| Spermidine | 0.033397 | 1.66981 | 1.53829 | up |
| Betaine | 0.0463947 | 1.62412 | 1.49564 | up |
| Propylparaben | 0.00541794 | 1.57631 | 1.80117 | up |
| Val-Gly | 0.0420337 | 1.52623 | 1.48169 | up |
| 5,6-Dihydro-5-Methyluracil | 0.00508626 | 1.52381 | 1.79113 | up |
| Acetylvaline | 0.0241531 | 1.52377 | 1.58862 | up |
| 12,13-EpOME | 0.0280335 | 1.51905 | 1.57409 | up |
| 11,12-EET | 0.0267652 | 0.600274 | 1.59975 | down |
| 20,26-dihydroxyecdysone | 0.0231703 | 0.576258 | 1.59608 | down |
| 12-HHT | 0.0115292 | 0.545391 | 1.72295 | down |
| D-Melezitose | 0.0460217 | 0.409577 | 1.49451 | down |
| Maltotriose | 0.0460217 | 0.409577 | 1.49451 | down |
| Raffinose | 0.0460217 | 0.409577 | 1.49451 | down |
| p-Cresol glucuronide | 0.00637241 | 0.161445 | 1.78844 | down |

Supplementary table S5. CAP2 vs CAP4

| Table S5: Metabolites significantly different between CAP2 and CAP4 | | | | |
|---|---|---|---|---|
| CAP2 VS CAP4 | P value | Fold change | VIP | Trend |
| 1,5-Diaminopentane | 0.0257273 | 3.4142 | 1.68526 | up |
| N-Methyl-α-aminoisobutyric acid | 0.045333 | 2.91262 | 1.5321 | up |
| 9,10-Epoxy-18-hydroxyoctadecanoic acid | 0.0268224 | 2.28103 | 1.63316 | up |
| N-Acetyl-L-Leucine | 0.0420605 | 2.08222 | 1.47608 | up |
| Val-Gly | 0.0273168 | 1.76672 | 1.44553 | up |
| Orotic Acid | 0.0108093 | 1.60807 | 1.76536 | up |
| Inosine | 0.00521438 | 1.56735 | 1.8426 | up |
| Lys-Asn | 0.0219421 | 1.53684 | 1.63494 | up |
| Cycloleucine | 0.00816947 | 0.665796 | 1.83287 | down |
| Ile-His | 0.0200466 | 0.663335 | 1.77131 | down |
| Tripropylamine | 0.00154698 | 0.639782 | 1.85224 | down |
| 5-Oxoproline | 0.0273181 | 0.639363 | 1.7201 | down |
| 7-Ketocholesterol | 0.00259514 | 0.584273 | 2.02057 | down |
| Colneleic acid | 0.028829 | 0.566018 | 1.43296 | down |
| 20,26-dihydroxyecdysone | 0.016415 | 0.54991 | 1.82764 | down |
| p-Cresol glucuronide | 0.00483603 | 0.141213 | 1.90938 | down |

Supplementary table S6. CAP3 vs CAP4

| Table S6: Metabolites significantly different between CAP3 and CAP4 | | | | |
|---|---|---|---|---|
| CAP3 VS CAP4 | P value | Fold change | VIP | Trend |
| D-Melezitose | 0.0151042 | 3.51279 | 1.96879 | up |
| Maltotriose | 0.0151042 | 3.51279 | 1.96879 | up |
| Raffinose | 0.0151042 | 3.51279 | 1.96879 | up |
| Orotic Acid | 0.00636939 | 1.58477 | 2.14848 | up |
| 2-Hydroxy-6-Aminopurine | 0.0357419 | 0.622645 | 1.84995 | down |
| Guanine | 0.0357419 | 0.622645 | 1.84995 | down |
| Spermine | 0.0296401 | 0.615434 | 1.80837 | down |
| Ala-Tyr | 0.0217337 | 0.568712 | 1.86341 | down |
| Pro-Gln | 0.032093 | 0.408944 | 1.43143 | down |
| 5-Hydroxyindole-3-Acetic Acid | 0.0151661 | 0.311204 | 2.10953 | down |
